# The BrainHealth Project study protocol: a scalable digital approach to measuring and promoting multidimensional brain health across the lifespan

**DOI:** 10.3389/fnhum.2025.1678165

**Published:** 2025-12-08

**Authors:** Lori G. Cook, Jeffrey S. Spence, Erin E. Venza, Aaron Tate, Ian H. Robertson, Mark T. D’Esposito, Geoffrey S. F. Ling, Jane G. Wigginton, Sandra Bond Chapman

**Affiliations:** 1 School of Behavioral and Brain Sciences, Center for BrainHealth, The University of Texas at Dallas, Dallas, TX, United States; 2 Trinity College Dublin, Global Brain Health Institute, Dublin, Ireland; 3 Trinity College Institute of Neuroscience, Dublin, Ireland; 4 Department of Molecular and Cell Biology, Helen Wills Neuroscience Institute, University of California, Berkeley, CA, United States; 5 Department of Neurology and Neuroscience, School of Medicine, Johns Hopkins University, Baltimore, MD, United States

**Keywords:** brain health, cognitive training, digital health, executive function, healthy aging, intervention, lifestyle, neuroplasticity

## Abstract

**Background:**

Optimization of brain health is a focal point in medical science, yet data regarding measuring, improving, and preserving lifelong brain health are lacking. This void demands an objective, change-sensitive measure of brain health and proven strategies to strengthen brain performance. The BrainHealth Project addresses these key issues, drawing upon neuroplasticity evidence of persistent modifiability of brain function across the lifespan. This landmark study aims to (i) evaluate and refine multidimensional change indices of brain health, (ii) evaluate the impact of evidenced-based cognitive strategies and lifestyle interventions on improving/maintaining brain health, and (iii) elucidate the mechanisms associated with brain health gains/losses.

**Methods:**

This quantitative, longitudinal, interventional, open-label, single-arm clinical trial aims to recruit 100,000 generally healthy adults ages 18–100 and evaluate changes for 10 years or longer. Assessments, coaching, and training are conducted online through the secure BrainHealth Platform, allowing utilization tracking. The BrainHealth Index (BHI) – a multidimensional assessment – is offered at baseline and every 6 months. Participants have access to coaching every 3 months and continual access to self-paced trainings and resources. The primary outcome is the composite BrainHealth Index score and its empirically derived subdomain scores: Clarity (cognitive health), Connectedness (social health), and Emotional Balance (mental health). The BHI includes validated measures sensitive to change, including gains or losses associated with behavior change and integration of cognitive strategies into daily life. This Index contrasts with traditional assessments focused primarily on detecting cognitive decline or diagnosing pathological conditions, yet it, too, has been found to be sensitive to small incremental losses. The primary online training, SMART (Strategic Memory Advanced Reasoning Tactics), is a strategy-based program validated with 25+ years of research. SMART promotes improvements in neural health, cognition, wellbeing, connectedness, and real-life function, previously reported in randomized controlled trials (RCTs). Statistical approaches focus on individual prediction using nonlinear models trained with large samples and on assessing mechanisms influencing gains or losses on brain health metrics.

**Discussion:**

This research extends prior RCT evidence to a longitudinal, epidemiologic approach, leveraging digital health and machine learning tools to deliver a generalizable measure of longitudinal brain health and actionable ways to achieve precision brain health. By integrating advanced statistical methods and large-scale data, the BrainHealth Project aims to provide medicine and society with accurate, multidimensional, sensitive, and actionable ways to optimize brain health practices across the lifespan.

## Background

Improving brain health is one of the most critical health challenges facing global society. The brain, as the command center for all our actions, feelings, and thoughts, influences every aspect of our life. Yet, the importance of optimizing brain health has been historically overlooked, in part due to outdated beliefs that intellect is fixed and that the brain cannot improve after young adulthood. However, neuroscientific discoveries over the past few decades have demonstrated that the brain retains neuroplasticity across the lifespan, meaning it can be shaped by experience and use at every age. While starting earlier in life offers greater opportunity to optimize brain performance, evidence shows that meaningful change remains possible across the lifespan (Merzenich and De Charms, 1996; Merzenich et al., 2014).

This understanding drives the need for objective ways to (i) measure changes in brain performance, whatever one’s starting point, and (ii) examine the array of habits and practices that can strengthen brain systems and performance at an individual level. Drawing on parallels with well-established preventative heart health strategies, this study hypothesizes that brain health care practices are optimized by initiation well before a person experiences losses in cognitive, psychological, or social function. A healthier brain could provide protective benefits against a range of neurological, psychiatric, age-related and behavioral challenges (Sabayan et al., 2025). Moreover, such benefits would, if successful, provide tremendous personal, social and economic benefit (Greene, 2021), extending peak brain performance well beyond the late twenties to early thirties, when documented losses have been found to begin (Hartshorne and Germine, 2015; Lu et al., 2011).

We define brain health as the integrated functioning of cognitive, emotional, and social processes. Efforts to keep the brain healthy are achieved by the continual optimization of these three dimensions. As such, promoting brain health will support a person’s ability to more fully realize their potential over the life course (World Health Organization, 2022).

Historically, widely used instruments for measuring brain health have relied heavily on disease-driven metrics that emphasize documenting decline and detecting problems. These tools have tended to focus narrowly on cognitive function, overlooking the complex inter-relatedness of cognitive, emotional and social functioning in a person’s life. Moreover, by prioritizing focus on detecting decline, not improvement, such measures may inadvertently reinforce stigma associated with brain issues and limit the ability to capture positive changes in brain health driven by mutually reinforcing gains across cognitive, emotional and social domains.

What has been missing is a health- rather than illness-focused approach. Measuring and monitoring brain performance through a health lens could open the way to novel, prevention-oriented interventions. Tracking gains in brain health would offer a viable foundation for transforming the public’s understanding of brain health – comparable to the shift which occurred for heart health in previous decades.

The BrainHealth Project’s longitudinal design will track gains as well as decline in a uniquely multi-dimensional way. Recent research shows that it is possible to enhance both neural (fMRI and MRI) and behavioral (cognitive, emotional and social) aspects of brain health (Chapman et al., 2015, 2016, 2017; Vas et al., 2016). Based on these results, we hypothesize that otherwise healthy adults without identified brain disease can improve their brain health and performance across the lifespan, when provided tools to establish healthy brain habits. The current protocol entails: (i) measuring and monitoring brain health changes over time; (ii) providing easy access to validated training protocols and refinement of personalized tools aimed at improving: executive functioning, stress management, social connectedness and associated healthy lifestyle choices (Chapman et al., 2019).

The BrainHealth Project was inspired by the paradigm-shifting Framingham Heart Study, which since 1948 has transformed the world’s understanding of cardiovascular disease by identifying modifiable risk factors and providing crucial insights into the prevention, diagnosis and management of heart disease and stroke (Andersson et al., 2019; Bitton and Gaziano, 2010). The BrainHealth Project aims to do something similar for the brain by identifying protective factors that can lead to proactive, preventive strategies to improve brain health. The aim is to achieve for brain health what was so successfully achieved in heart health globally via a health/wellness- rather than pathology/disease-focus.

The measures used in this protocol as well as the interventions offered arose from a decades-long program of research at The University of Texas at Dallas’ Center for BrainHealth, led by cognitive neuroscientist Dr. Sandra Chapman, PhD (Principal Investigator). The holistic, three-dimensional primary outcome measure for the study is the BrainHealth Index (see below). The interventions were based on a series of Phase 2 randomized trials. Central to the interventions offered is a well-validated, strategy-based training protocol known as SMART (Strategic Memory Advanced Reasoning Tactics), which has been shown to promote real-life cognitive gains, strengthen key executive and innovation networks in the brain and promote improved planning, decision-making, judgment and emotional regulation (Chapman and Mudar, 2014).

Building on prior work, this study aims to translate prior in-person brain health assessment and training protocols to an online, widely accessible portal via the BrainHealth Platform. This scalable, secure portal allows scientists and participants to continuously track engagement and visually see the impact of their adoption of new habits given brain health strategies, coaching, and education over time.

Therefore, the primary objectives of the BrainHealth Project are to:

i    Assess factors associated with brain health and performance over the adult lifespan using the multidimensional BrainHealth Index to measure change - whether gains or losses - in healthy adults.
ii    Examine the impact of evidence-based cognitive strategies and lifestyle interventions on brain health changes using machine learning.
iii    Elucidate the mechanisms associated with brain health gains or losses.

This third objective is being addressed in a BrainHealth imaging sub-study protocol (currently underway). The goal of this sub-study is to assess the neural and biological mechanisms associated with, or predictive of, improved or maintained brain health through changes observed in fMRI brain scans collected at baseline, 6 months, and annually thereafter for the duration of the study. The neural, mechanistic data from this sub-study will be analyzed alongside the BrainHealth Index behavioral data to help develop a reliable set of biomarkers for assessing brain health longitudinally.

By integrating large-scale data across multiple domains and time intervals, the aim is to evaluate, interpret, and predict how various factors interact to contribute to brain health at both group and individual levels. These will be assessed through an epidemiological approach, utilizing interventions previously shown beneficial in prior randomized trials (Chapman and Mudar, 2014; Chapman et al., 2015, 2016, 2017; Vas et al., 2016). Due to the open-label design of the study, all participants have access to interventions found previously to promote benefit, without harm. The data from this large, diverse population study will provide granular insights into factors such as involvement, health, and lifestyle, enabling the development of prediction models at the individual level. We predict dose-dependent response, in that participants who consistently engage in the micro-learning modules from the online BrainHealth Platform training over time will show improved and sustained brain health outcomes compared to those who do not.

In sum, the BrainHealth Project aims to better understand how scalable, validated assessments and interventions can effectively leverage neuroplasticity to enhance brain health at any age, encouraging people to adopt habits and practices to continuously strengthen their brain performance to thrive in their context. Through better understanding of factors that optimize brain health across the lifespan, the potential for improved health, work, and economic-related outcomes would provide considerable value to both the individual and society at large (Greene, 2021).

## Methods

### Study design

The BrainHealth Project is a quantitative, longitudinal, interventional, open-label, single-arm clinical trial. This design was chosen because the component interventions have been previously validated in a series of randomized-design studies (Chapman and Mudar, 2014; Chapman et al., 2015, 2016, 2017; Vas et al., 2016). A pilot study was conducted from March to August 2020 to assess the feasibility of the online study platform and to develop a data-driven index of brain health (Chapman et al., 2021). The study aims to enroll 100,000 adults over a 10-year period, with ongoing recruitment and enrollment throughout the study duration. As the study progresses, data analysis and findings will inform the addition of more targeted data collection, and both interventional and observational sub-studies are planned.

All core procedures for the Project are conducted online via the BrainHealth Platform, accessible via web, iOS, or Android applications through a personal computer or a full-function mobile device (native app launched in February 2024). Participants complete an initial online baseline assessment of their BrainHealth Index, followed by personalized online coaching every 3 months, with continuous access to online training, exercises, and educational resources aimed at optimizing brain health and overall wellbeing. Participants are free to choose their own level of engagement in training (e.g., engaging with the habit utilization but not the learning modules). Such utilization is tracked to evaluate the impact of engagement on measured brain change from baseline over time. Participants are encouraged to reassess their BrainHealth Index every 6 months for the duration of their involvement.

For a subset of participants who choose to link data from their personal fitness devices, metrics such as physiological markers related to sleep, physical fitness, and overall health will be collected. Participant engagement with the features and content available on the BrainHealth Platform will also be analyzed to assess the impact of cognitive interventions and the use of digital health and educational tools.

### Participants

Recruitment for the longitudinal study commenced in September 2020, following the conclusion of the pilot phase. The study targets generally healthy adults aged 18 years and older. Inclusion criteria focus on participants’ ability to access and engage with the online platform and content, as determined by self-report. These criteria include: fluency in English, access to an internet connection and device, and the ability to hear and read information on the computer or device. Exclusion criteria are based on specific health conditions. Individuals are excluded if they have a diagnosed neurodegenerative disease; a history of stroke, concussion, or brain injury that currently impairs their ability to conduct activities of daily living (ADLs); or a diagnosis of autism spectrum disorder with non-independent functioning. Participants are not excluded for other diagnoses, such as learning disorders or psychiatric or medical conditions, in order to maintain the diversity of the generally brain-healthy population and to ensure the generalizability of the results. These characteristics will be captured through self-report, along with changes in demographic data collected at each assessment timepoint, to inform potential clinical subgroup analyses as appropriate.

### Recruitment

Participants are recruited through word of mouth, online postings, social media and advertisements, such as through the Center for BrainHealth website and e-newsletter and registries such as the Alzheimer’s Prevention Registry as well as posts on social media platforms such as Facebook, Instagram, and LinkedIn. Study flyers or recruitment information are also shared through in-person and virtual events by the Center for BrainHealth and our healthcare and community partners. Participants do not receive any payment or reimbursement for participation in this online study.

### Data collected and instruments used

#### BrainHealth Index (BHI) assessment

BrainHealth Index (BHI) assessment (60–90 min total, avg. 70 min). The study’s primary outcome measure is the composite BrainHealth Index (BHI) score. The BHI is scored from a multidimensional assessment of brain health and performance, comprised of an online battery of cognitive performance measures and self-report questionnaires completed by participants through the BrainHealth Platform. The BHI is unique in that it is designed to measure change over time without the presumption of decline or a specified ceiling – i.e., characterizing the development and potential upward improvement of brain health rather than serving as a decline-oriented, adjusted normative, or diagnostic cognitive measure. Cognitive assessment includes tasks of complex thinking capacities such as reasoning, abstraction, mental flexibility, and strategy – each with alternate-stimuli versions randomized across timepoints. The self-report questionnaires measure other aspects of daily life that relate to brain health, such as emotional well-being, quality of life, purpose, happiness, resilience, social support systems, and sleep – utilizing tools that have been empirically validated in their respective literatures where possible. In these sections of the online assessment battery, participants self-report through rating aspects of their daily life within the prior 6 months. At each testing time point (baseline and every 6 months thereafter), participants have the opportunity to divide the assessments into shorter segments over time while saving their completed progress to date (for up to 2 weeks duration) to complete the overall assessment. See Table 1 for a full list of measures.

**TABLE 1:** Measures included in the multidimensional BrainHealth Index assessment.

Measure	Assessment instrument
Strategic attention	Visual Selective Learning Task (Hanten et al., 2007)
Abstraction	Proverb Interpretation Task [developed at the Center for BrainHealth, modeled after Uekermann et al. (2008)]
Reasoning	Test of Strategic Learning (TOSL) (Vas et al., 2015a,b)
Synthesis	Condensed synopsis of complex text (∼550-word narrative)
Interpretation	Fluency of take-home messages/interpretations from text
Memory	Memory for text details (free and cued/elaborated recall)
Innovation	Fluency of high-level interpretations from picture interpretation task [developed at the Center for BrainHealth, modeled after semantic verbal fluency task, adapted from Lezak et al. (2004)]
Processing speed	Coding/Digit Symbol Task [modified from the Digit-Symbol Verification Task – DSVT (Rypma et al., 2006)]
Sleep	Pittsburgh Sleep Quality Index (PSQI) (Buysse et al., 1989)
Compassion	Questionnaire adapted from the Light Triad Scale (Johnson, 2018; Strauss et al., 2016)
Mood Depression Anxiety Stress	Depression Anxiety Stress Scale (DASS-21) (Lovibond and Lovibond, 1995)
Meaningful activities/purpose	Engagement in Meaningful Activities Survey (EMAS) (Eakman, 2011)
Happiness	Oxford Happiness Questionnaire (OHQ) (Hills and Argyle, 2002)
Social support	Social Support Survey Index (Sherbourne and Stewart, 1991)
Resilience	Connor-Davidson Resilience Scale (Connor and Davidson, 2003)
Life satisfaction	Quality of Life Scale (Burckhardt and Anderson, 2003)
Social engagement	Social BrainHealth Scale (developed at the Center for BrainHealth) (Allen et al., 2020)
Growth mindset	BrainHealth Appraisal Questionnaire [developed at the Center for BrainHealth based on Bandura (2006)]
Fitness	Metabolic Equivalents: Cardiorespiratory Fitness (CFEQ) (Jurca et al., 2005)

**FIGURE 1:**
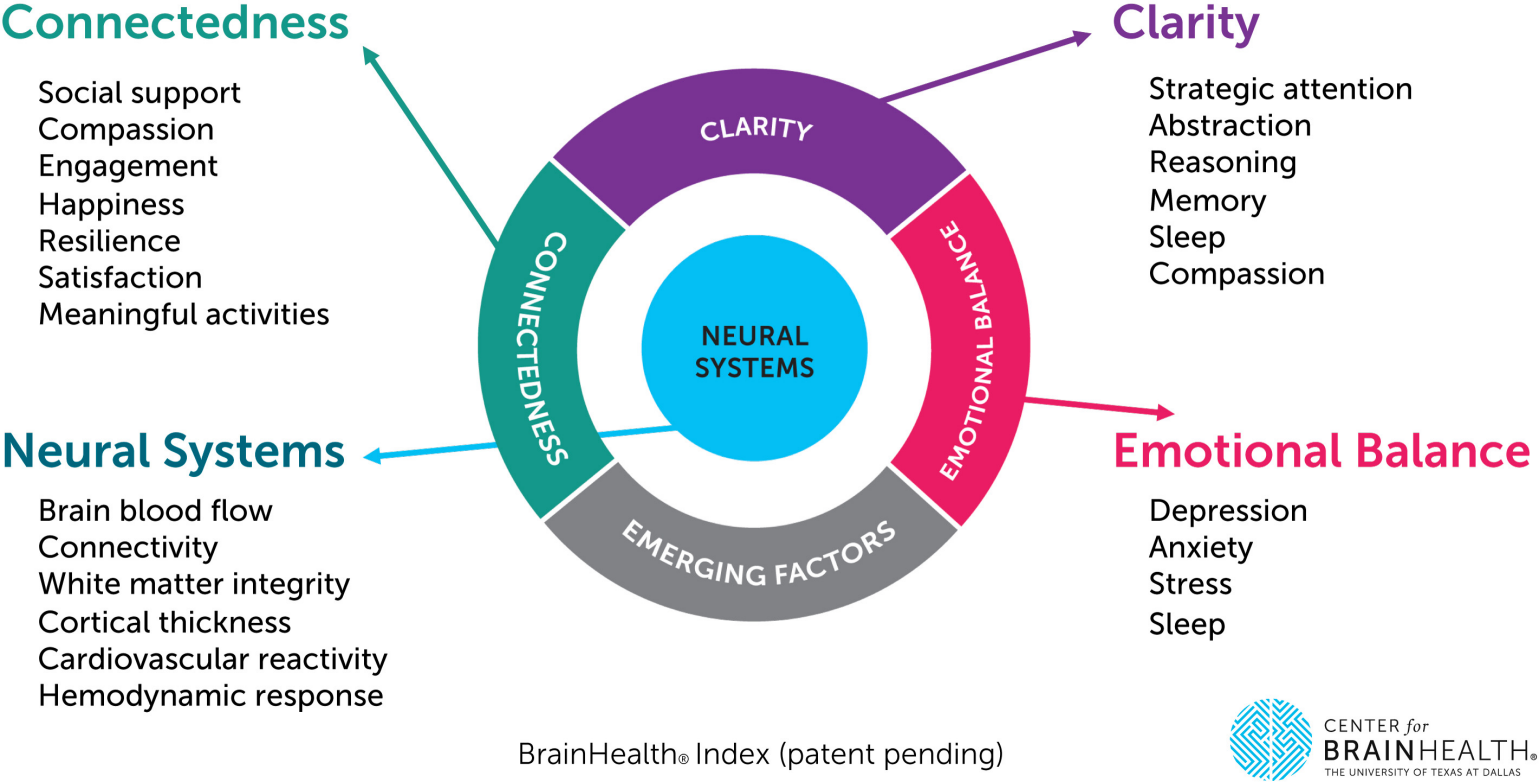
The BrainHealth Index provides a holistic measure integrated across three empirically derived subdomains (clarity, connectedness, and emotional balance). This figure depicts each subdomain area as well as their corresponding measures (including neural metrics to be collected as part of a separate phase/subset of the BrainHealth Project protocol).

The BrainHealth Index yields four separate scores which are shared with participants through their BrainHealth Platform study dashboard, including a composite/global BHI score as well as scores for its three empirically derived subdomains: (1) Clarity (readiness to reason through complex situations and create new opportunities or solutions, i.e., cognitive health), (2) Connectedness (to people and purpose, i.e., social health), and (3) Emotional Balance (steadiness in the face of difficult situations, ability to handle adversity while remaining productive and capable). These are based on a factor analysis of change scores from measures shown in Table 1 (Chapman et al., 2021). The University of Texas at Dallas’ Center for BrainHealth investigator team designed the BHI’s composite/holistic score to capture the multiple dimensions of brain health and the interdependency amongst these dimensions, based on machine learning analytics, rather than presupposed components. For each individual, the four scores are graphed to visually track progress over time. This data visualization allows participants to realize their brain health as a whole (composite BHI score) while also reflecting the multiple paths they have to support their brain health and performance over time (factor subdomain scores). The BHI uses the individual’s own performance against which to measure growth over time rather than being compared to a group norm, allowing for a more personalized approach that is independent of starting point. See Figure 1, which displays the three subdomain areas and their contributing measures.

#### Demographic information

For each participant, self-report data on age, gender identity, biological sex assigned at birth, race/ethnicity, country/U.S. zip code, household income, level of education, and occupation as well as any currently diagnosed medical or psychiatric conditions is collected at baseline. At every subsequent online assessment timepoint (offered every 6 months), any changes in these data points are also reported.

### Training materials

The online training materials included in this study protocol consist of the following:

#### BrainHealth platform training modules, habits, and resources

##### Training modules

Participants access the training modules through the study’s online BrainHealth Platform which includes micro-learning videos, animations to motivate learning, activities, and learning/strategy application opportunities (Chapman et al., 2021). The training protocol is comprised primarily of evidence-based cognitive strategy learning, previously shown to improve aspects of neurocognitive and real-life function in clinical trials (Chapman and Mudar, 2014; Chapman et al., 2015). The strategy-based cognitive training protocol, Strategic Memory Advanced Reasoning Tactics (SMART), was developed by Center for BrainHealth neuroscientists and clinicians to promote improvement in executive functioning and self-agency (Chapman, 2014). The executive function/top-down SMART protocol trains three core strategies, specifically (1) *strategic attention* to reduce information intake, single-task to focus and make progress on daily goals, and brain down time, (2) *integrated reasoning* to quickly synthesize information/facts into succinct abstracted ideas and interpretations and apply to real life application, and (3) *innovation* to flexibly generate a multitude of ways to improve tasks, communications, meetings, relationships and missteps/mistakes. The strategies can be applied in the context of everyday activities and responsibilities. The efficacy of SMART has been evaluated in a range of healthy and clinical populations across the lifespan. These previous clinical studies of SMART demonstrated (i) cognitive improvement in aspects of executive function and memory (Cook et al., 2014; Mudar et al., 2017; Vas et al., 2011, 2016; Venza et al., 2016), enhanced processing speed (Motes et al., 2018), and academic achievement (Gamino et al., 2014, 2022) as well as (ii) improved psychological well-being including reduction of depressive, stress, and anxiety symptoms (Vas et al., 2016). Gains in these areas were found to correspond with significant neural changes, including aspects of neural connectivity, cerebral blood flow, and neural efficiency (Chapman et al., 2015, 2016, 2017; Gallen et al., 2016; Han et al., 2017, 2018; Motes et al., 2018; Yang et al., 2018).

All BrainHealth Platform SMART sessions are designed to be self-paced, consisting of 5- to 10-min daily units. These sessions are reinforced with habit training and integration, encouraging participants to consistently apply tactical brain strategies to their various everyday responsibilities and situations. Although training is available daily, participants can determine their own frequency of engagement. The training units combine education on brain strategies, explanations of the rationale to promote brain health literacy, and prompts for personal reflection and practical application. After completing the four SMART modules, training continues with online learning about solutions to minimize stress. These SMART strategies associated with stress management techniques are designed to build a more resilient mindset by integrating stress management techniques with healthy lifestyle choices such as physical exercise, diet, and mindful meditation – practices that have been empirically shown to reduce stress (Chow, 2020; Esgunoglu et al., 2021; Khoury et al., 2013; Owen and Corfe, 2017; Penedo and Dahn, 2005). The sleep module provides information on the science of sleep and aspects such as sleep cycles, as well as covers sleep hygiene tips to help improve their quality and quantity of sleep. For detailed descriptions of these self-paced BrainHealth Platform training modules, see Table 2.

**TABLE 2:** Description of self-paced online training modules.

Training module	Description	No. of units/total time
1. SMART 01*	Provides strategies and interactive activities teaching how to block irrelevant information and focus on key priorities and critical information (strategic attention). Example: Organize your day to accomplish significant tasks – each day prioritize the top two tasks that require deeper-level thinking.	6 units/55 min
2. SMART 02*	Provides strategies and interactive activities on how to abstract big-picture concepts from information to better inform understanding and real-life decisions (integrated reasoning). Example: Extract key concepts from incoming information vs. trying to process and remember everything.	4 units/35 min
3. SMART 03*	Provides strategies and interactive activities on how to generate multiple and diverse solutions/perspectives/questions to strengthen mental flexibility (innovation). Example: Identify multiple alternative perspectives/ideas on discordant issues.	6 units/35 min
4. SMART 04*	Provides real-life application scenarios where participants can practice dynamic implementation of the strategies from SMART 01–03 (strategic attention, integrated reasoning, innovation) in a synergistic manner. Example: Think about and prepare to ask your boss for a raise (considering your accomplishments, impact those accomplishments have had or could have on the organization, etc.).	6 units/45 min
5. Stress solutions 01	Presents information about physiological and neurological response to stress, as well as cognitive strategies linked with SMART to manage and reframe stressors. Example: Reframe your perception of your response to a difficult situation from anxiety to excitement.	5 units/40 min
6. Stress solutions 02	Provides accessible techniques to help “recharge your mental battery” in terms of stress or fatigue, as well as education on lifestyle factors that can positively impact overall health. Example: Take several short breaks throughout your day.	4 units/30 min
7. Stress solutions 03	Provides research on the benefits of mindfulness, meditation, and healthy sleep habits, as well as practical tips on how to practice each one (linking with SMART strategies). Example: Participate in a mindfulness exercise.	5 units/45 min
8. Sleep	Presents research on the science behind sleep over the lifespan, the brain impacts of poor sleep, and practical tips for improving one’s sleep habits.	16 units/75 min
Total time		350 min

*Modules 1–4 provide the foundational cognitive strategies (SMART).

##### Habits

Each training module is paired with a set of brain-healthy “Habits” that participants can access once they complete the module. These habits allow participants to integrate specific training concepts or strategies into their daily routines. Participants can choose a habit, opt to receive daily reminders, and track their progress over time, earning levels and digital “badges” on the BrainHealth Platform. Examples of habits include minimizing distractions, taking regular breaks, completing two important big tasks each day, maintaining a consistent and healthy sleep routine, and exploring multiple possibilities to reapproach a challenge, perceived failure, dilemma or mistake. Additionally, habits can be focused on lifestyle improvements such as exercise, nutrition, and mindfulness.

##### Challenges

After completing the primary training modules, participants advance to a continuing series of “Challenge” units designed for reinforcement and extended application of the strategies and practices. These challenges are also delivered in 5- to 10-min increments and feature diverse content, such as article readings and educational video clips. Each provides insights into the relevant brain science and includes reflection questions or practical application opportunities. Monthly challenge topics are organized into four weekly segments centered around a shared theme. Examples of these themes include memory habits, managing news consumption, navigating tough conversations, the science of confidence, breathing techniques, gratitude practices, etc.

##### Resources

Participants have access to a continually curated collection of educational resources on the broad topic of brain health, including media articles, published research studies, online lectures, etc., Additional training and resource content will be added throughout the study to offer ongoing learning opportunities and maintain participant engagement.

### Online coaching

Participants can engage in individual 20-min videoconference coaching sessions every 3 months, conducted within the study platform. These sessions are led by a study brain health coach and can be self-scheduled by participants. Coaches are assigned based on availability and consist of study personnel with degrees spanning fields such as speech-language pathology, psychology, occupational therapy, education, or other areas of human performance. All coaches have experience with the BrainHealth Index and are trained in administering and interpreting cognitive and human performance assessments. Each has a minimum of 2 years of post-graduate work experience in their respective field of practice.

During these sessions, brain health coaches offer individualized feedback on participants’ BrainHealth Index results, guide them on engaging with the online training content, assist in setting personal brain health goals, and discuss how to apply the training strategies and practices to their specific goals or context. Following the session, coaches provide summary notes on the participant’s BrainHealth Platform profile, articulating the key points discussed for easy access at any time. Participants can choose to utilize this quarterly personalized coaching as much or as little as they prefer.

### BrainHQ training

After completing their third BrainHealth Index assessment (typically 1 year post-baseline), participants have the opportunity to access BrainHQ (2024) training for a limited time. Through the BrainHealth Platform, participants can engage in 14 BrainHQ training exercises over a period of up to 16 weeks. In contrast to SMART, which is strategy-based, BrainHQ is a computer exercise-based approach to cognitive training, with multiple studies supporting its effectiveness in enhancing cognitive performance (Smith et al., 2009). Each exercise adapts in difficulty according to the participant’s performance, using a specialized algorithm. The exercises made available to participants in the BrainHealth Project include those addressing attention, processing speed, memory, and decision-making. During the 16 weeks of BrainHQ access, participants are encouraged to engage with BrainHQ exercises four times a week for 30 min each session.

## Retention of participants

Recognizing the importance of engagement and retention in a longitudinal study, the research team has developed and implemented solutions to address participant engagement and retention through three avenues: (1) technology platform enhancements, (2) improved communications, and (3) opportunities for community building among participants.

Initially, the BrainHealth Platform was accessible only via a web browser-based interface. However, to enhance accessibility and user experience, it has evolved to include a dedicated mobile application, launched in February 2024. This mobile app introduces advanced notification capabilities such as optional push notifications, sounds, and banners, which not only prompt participants to re-engage after periods of disengagement, but also remind them to complete essential study tasks, like the BrainHealth Index assessments. Furthermore, gamification elements, such as earning “coins” for task completion and achieving higher ranks with each BrainHealth Index completed, have been added to increase participant utilization and motivation.

**FIGURE 2:**
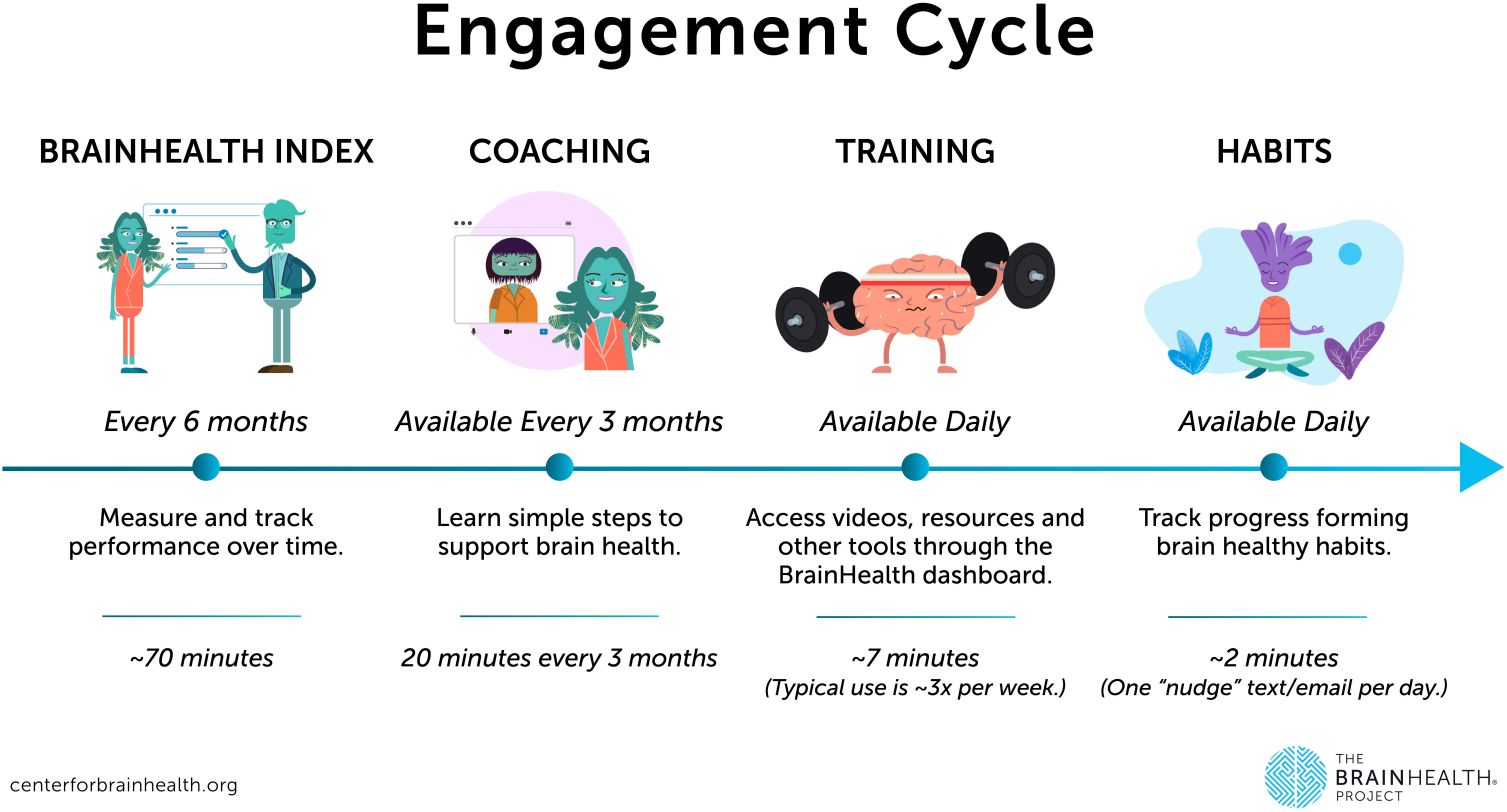
Visual outline of participant study engagement procedures.

Study communications have been continuously improved, primarily by making messaging clearer and more concise, ensuring participants are well-informed about their progress throughout the study procedures. The improved BrainHealth Platform now includes an onboarding navigation process that clearly outlines each study step, accompanied by visual tutorial elements. These tutorials help orient participants to their online study dashboard and explain or highlight key features to enhance ease of use. For a visual representation of the study participation elements, see Figure 2. Additionally, communication methods have been expanded beyond email, incorporating the use of text messaging for participants who opt-in. This allows for more regular and continuing study engagement, such as facilitating daily habit completion and other study interactions.

The BrainHealth Project team also works to enhance participant engagement by offering monthly virtual group coaching sessions via Zoom. These 45-min sessions are conducted live (not recorded) and are open to all study participants, providing social motivation and community-building opportunities. Additionally, in response to early participant requests for more information on the brain science behind the study, the research team hosts periodic virtual town hall webinars. These webinars, generally held annually or semi-annually, allow participants to deepen their understanding of the research they contribute to and foster a sense of involvement in a greater mission as citizen scientists. The town hall webinars are lecture-style, recorded, and subsequently made available in the BrainHealth Platform Resources section for all participants to access. Evaluation of the impact of these offerings will focus on whether these additional resources contribute to increased training utilization and improved study retention over time.

### Assessment scoring and quality control

The online BrainHealth Platform, which hosts assessments and training tools, automatically safely uploads data to a secure cloud-based data management system. The majority of the assessments included in the online BHI are automatically scored, except for three language-based cognitive performance measures (Abstraction, Reasoning, Innovation), which are manually scored by trained study personnel. To ensure data integrity and inter-rater reliability, quality control practices are in place. These include procedures for scoring review and consensus-building, as well as procedures for identifying and filtering invalid responses. This might involve noting any difficulties or interruptions that participants report encountering during the tasks or detecting the use of external aids.

### Statistical considerations

A key objective of the BrainHealth Project is to achieve personalized precision brain health, recognizing that lifestyle interventions, cognitive training, exercise, and sleep impact individuals differently. The ultimate goal is to improve brain health for all participants, yet this improvement requires a tailored approach to meet each individual’s specific needs. Consequently, the statistical approaches employed in this study prioritize individual prediction over population-wide inference. As such, formal power analyses are not relevant to sample size determination for the main study; however, as outlined below, some population-based sub-studies do require power justification.

In the machine learning literature, it is necessary to utilize tens of thousands of samples to train, validate and test complex, nonlinear prediction models such as neural networks (including deep learning networks), support vector machines and unsupervised tree or clustering methods. To determine the factors that influence and improve brain health in individual participants, these nonlinear models will be utilized, trained using large samples. Large samples will accommodate not only the training of the models, but also the validation and testing of them on independent samples such that estimates of generalization error can be obtained with low variance. Recruitment of a baseline sample of engaged participants is planned in order to yield robust data on at least 15,000–20,000 individuals over a 10-year period. These sample sizes are also comparable to those in the landmark Framingham Heart Study, which continues to identify critical factors that inform clinical practice and guide public education on heart health (Andersson et al., 2019; Bitton and Gaziano, 2010).

Increased attrition and lack of adherence have been longstanding challenges to successful health-behavior interventions (Middleton et al., 2013) and we fully anticipate that in the present study that meets people where they are. Even pharmaceutical prescriptions for common physical conditions such as psoriasis and arthritis have adherence rates of 30%–35% after 1 year (Hichborn et al., 2018). Hence, given the online nature of the study and precedent from previous longitudinal, population-based studies using internet-based/eHealth platforms (Eysenbach, 2005), an attrition rate of as much as 80%–85% over the full 10-year study period is anticipated.

Accordingly, recruitment efforts will aim to enroll a baseline sample of 100,000 participants to achieve the desired data yield.

In addition to the sample requirements of neural network prediction models, a sample of 15,000–20,000 enables population-based sub-studies as secondary aims in the BrainHealth Project. For example, three sub-studies – focused on workplace, mental illness, and caregiver groups – will analyze mean longitudinal changes in brain health. Potential declines in brain health that may occur due to unexpected health events as reported by participants, such as undergoing chemotherapy, experiencing a concussion, encountering significant grief, and other impactful medical or life events, will also be documented. Importantly, the capacity for recovery will be examined, particularly in relation to access to brain health training supports, as participants continue in the longitudinal study.

As the specificity of particular cohorts increases (e.g., males between 20 and 30 with less than a bachelor’s degree and a self-reported mental illness), the samples available for sub-analyses decrease markedly. However, having a total sample between 15,000 and 20,000 will allow rare cohorts to maintain at least samples of 100, which is required to detect effect sizes of at least 0.5 with 80% statistical power, adjusted for false positive control due to multiple testing.

## Discussion

This manuscript details the study protocol, background, and significance of the BrainHealth Project, a quantitative, single-arm, open-label, longitudinal interventional clinical trial. While significant research has historically been focused on improving cardiovascular health—thereby greatly enhancing life expectancy and quality—to date, comparatively limited efforts have been made to comprehensively assess and strengthen brain health in generally healthy individuals across all ages and life stages. The BrainHealth Project aims to address this critical gap by taking a multidimensional approach to measure, monitor, and enhance brain health in healthy adults, from older teens through late life. The study utilizes an online portal accessible via computers or mobile devices, to allow wide accessibility and to ensure scalability.

At the heart of this work, the BrainHealth Index fills a longstanding gap in medicine, research, and public health by providing a scalable, multidimensional framework that captures risks, gains, and resilience, advancing the capacity to measure and promote brain health throughout the lifespan. After completing their BrainHealth Index assessment, most participants express a desire to learn how they can actively strengthen the areas of brain health identified in their results. To meet this need, the BrainHealth Project builds on prior shorter-term, randomized control trials that demonstrated significant benefits from an in-person brain health intervention, known as Strategic Memory Advanced Reasoning Tactics (SMART). SMART, a manualized protocol focusing on executive functions and top-down strategies, showed gains from pre-to post-training across neural, cognitive, emotional, and social health domains, with improvements persisting at least three months post-training (Chapman et al., 2015, 2016, 2017; Vas et al., 2016). The current ongoing trial integrates SMART with technology and coach-facilitated lifestyle changes—including sleep, stress management, social connections, and physical exercise—to create a more comprehensive and scalable extension of the previously evaluated program. This trial aims to explore the long-term impacts of validated brain health interventions. A central aspect of this approach is the regular measurement of wide-spectrum brain health using regular validated BrainHealth Index assessments, including a subset of participants with comprehensive brain imaging measures over time.

Major strengths of the BrainHealth Project include a focus on the following seven issues that will inform ways to motivate behavioral change to improve brain health on a population-wide basis:

1. Utility of the BrainHealth index: Evaluating the BrainHealth Index’s ability to measure changes in *multidimensional brain performance*, where each participant serves as their own control. This approach charts individual changes over years across a multidimensional composite metric as well as changes demonstrated on the constituent factors. The individualized performance tracking is possible regardless of a person’s starting level and is independent of education, economic status, cultural background, or gender. This approach stands in contrast to conventional siloed assessments that rely on normed measures that focus on domains designed primarily to detect decline and deliver diagnoses and are adjusted for expected losses with age. Our research demonstrates that individuals can fall outside the so-called normal range and still achieve meaningful improvement, whether their baseline performance is very low or very high. Moreover, repeated administrations of the BrainHealth Index show no significant practice effects, even when administered over relatively short intervals such as 3 months or longer (Chapman et al., 2021; Young et al., 2025; Zientz et al., 2023).
2. Potential gains across all ages: Assessing the potential for adults of all ages, not just older adults, highlights the opportunity to build reserve and strengthen healthy brain habits when individuals are still in early to middle adulthood through simple top-down cognitive strategies of executive function. Cultivating these skills fosters a sense of self-agency that extends to other health habits and to broader aspects of life.
3. Long-term impact: Characterizing the long-term effects of brain health strategy and lifestyle interventions over multiple years through a longitudinal, epidemiologic-type approach. It also examines changes that may result from medical treatments, such as chemotherapy, hormone replacement therapy, and sleep interventions – whether positive or negative.
4. Digital health accessibility: Measuring the utility of leveraging digital health technology to enhance accessibility, reaching across urban to rural areas and extending far beyond major research centers or clinical specialists and providers.
5. Precision with machine learning: Utilizing machine learning tools to rapidly develop precision brain health at an individual analysis level that can provide personalized brain health scores and insights to set goals.
6. Mechanisms of brain health changes: Elucidating significant mechanisms underlying changes in brain health, including gains or losses, by enrolling large-sample, diverse cohorts.
7. Neural changes and neurobiological markers: Setting the stage for identifying neural changes through the BrainHealth Project’s subset imaging protocol (detailed in a separate manuscript). These imaging data will help link neurobiological markers that correspond to both gains and potential future losses in brain health, thereby offering mechanistic insight into the patterns already captured by the behavioral BrainHealth Index. Although MRI provides valuable mechanistic insights, its high cost and limited accessibility make it impractical for widespread use in population-level brain health monitoring. This study therefore focuses on scalable, validated proxy measures derived from the behavioral BrainHealth Index that can accurately, reliably, and reproducibly reflect and possibly predict changes in individual brain health across the lifespan.

In summary, the BrainHealth Project represents a concerted single-arm research study designed to test the feasibility and effectiveness of measuring and enhancing brain health using a previously validated assessment tool and science-backed interventions in an augmented and scalable manner. Brain health encompasses the optimization of neural health, cognition, connectedness to people and purpose, and emotional wellbeing across the lifespan – aligning with and expanding the World Health Organization’s definition of brain health (Hartshorne and Germine, 2015). These domains, except neural health, are measured biannually using a multidimensional measure - the BrainHealth Index. Each of the three factors (i.e., clarity, connectedness, and emotional balance) can be both a contributor to, as well as a consequence of, underlying neural health. The Project represents an innovative study “in the wild” – meaning that the online offerings allow the participants to partake of the protocols when and where they choose – without having to come to a specialty or research center. This design allows us to assess the degree of compliance and attrition over time, the latter of which is anticipated to be high given the well-documented tendency for individuals to struggle with sustained adherence, even for guidelines supported by strong evidence of benefit (Hichborn et al., 2018; Middleton et al., 2013). The strength of such a design is its real-life applicability, its scalability, and cost-effectiveness, given the very low human contact required.

The study also aims to elucidate the potential neural and other physiological mechanisms that contribute to changes in brain health. By analyzing brain and physiological data, the research will inform how various aspects of brain function—such as neuronal activity, blood supply, and metabolism—are influenced by interventions and captured by changes in the BrainHealth Index over time, presumably reflecting improved brain health. The ultimate goal of this project is to provide a clinically meaningful, widely accepted, generalizable measure of the brain that supports the development and testing of precision, preventive, and personalized brain health interventions, making them widely available globally.

The centrality of brain health to overall health underscores the potential of scalable interventions like the one described in this project to broadly impact public health more generally. Given that some brain health aspects of neural health and cognitive performance, such as global cerebral blood flow (Lu et al., 2011) and processing speed (Hartshorne and Germine, 2015), often begin to decline in early adulthood, this current project is relevant across the entire adult lifespan. It is applicable even in the absence of disease or injury and should be employed starting in early adulthood to foster and reinforce healthier brain habits across whole populations.

### Limitations

As with any longitudinal study, particularly one conducted entirely online, participant dropout is inevitable and expected, allowing dropout to be a factor to study. Another limitation is the potential for selective attrition, in that those who continue to participate beyond the baseline assessment to complete later assessment timepoints may also likely be those most highly motivated to succeed, particularly given possible gains after the initial training instruction period. Efforts will be made to recruit and retain more diverse populations (racial, level of education, socioeconomic status and others) over the course of the study to enhance study sample representativeness and support the generalizability of study findings.

This study lacks a randomized control group. However, the decision to adopt a single-arm study design was informed by findings from our prior randomized clinical trials demonstrating benefit of SMART in the majority of participants. Given the significant time commitment required from participants, it was deemed more ethically appropriate to use a scientifically valid design where each participant serves as their own control and stands to benefit from engagement in the study. Moreover, the large sample size and extended duration allow for the use of quasi-experimental designs and machine learning methods to monitor changes in brain health based on the extent of usage of the brain health interventions offered. This approach not only aligns with the goal of precision brain health but also allows for the consideration of individual differences such as medical history and lifestyle habits. These personal factors are systematically analyzed using machine learning models, which further enhance the ability to tailor interventions based on unique participant profiles and to more accurately predict outcomes based on diverse personal data inputs. Feasibility testing is underway for integrating machine learning and artificial intelligence tools to support manual scoring components and live, virtual coaching.

Finally, web-measured changes in brain health will be correlated with and validated against brain imaging-derived neural measures in a subset of participants. This correlation aims to strengthen the hypothesis that adopting healthy brain habits can yield long-term benefits to brain health, as behavioral changes over extended time periods that align with desirable neural changes are observed.

### Future directions

The BrainHealth Project aims to address critical clinical questions surrounding brain wellness and the potential for cognitive gains across the lifespan. As other online brain health interventions are validated through clinical trials, the BrainHealth Platform and the BrainHealth Index present opportunities for collaboration with researchers and medical experts who have evidence-based brain health protocols suitable for online implementation. By incorporating and testing these additional protocols and interventions, whether non-pharmacological or pharmacological, the efficacy of the BrainHealth Index as a robust outcome of these various interventions over time can be further evaluated. The goal is to eventually make the BrainHealth Platform and Brainhealth Index open access to the degree possible.

Currently, there is no widely accepted “gold standard” for a multidimensional measure of normal brain health. The BrainHealth Index serves to fill this void by offering an urgently needed standard that is sensitive to both meaningful positive and negative changes across key dimensions of brain health, including neural function, clarity of cognition, emotional well-being, and connectedness to people and purpose. By enhancing our understanding of the factors that optimize brain health throughout the lifespan, the BrainHealth Index has the potential to significantly impact health, research, work, and economic outcomes, benefiting individuals, science, and society as a whole.

### Conclusion

In summary, this groundbreaking study aims to decode the determinants of brain wellness and cognitive resilience across the lifespan. Drawing inspiration from the paradigm-shifting Framingham study and adopting a similarly broad, primarily observational approach, the BrainHealth Project seeks to build a comprehensive repository that advances understanding of how brain health can be maintained and enhanced beyond the traditional boundaries of the aging brain. Through rigorous longitudinal analyses and integration of diverse datasets, the overarching aim is to identify actionable strategies that strengthen brain wellness and cognitive performance in diverse populations. Ultimately, if successful, this effort could reshape the landscape of brain health research and clinical practice guidelines.
